# Prognostic Role of Copeptin, H‐FABP, and TTE in Pulmonary Embolism in the Emergency Department

**DOI:** 10.1155/emmi/9544238

**Published:** 2026-06-29

**Authors:** Huseyin Furkan Kucukbezirci, Seda Ozkan, Eser Durmaz, Fatih Cakmak, Ibrahim Ikizceli, Dildar Konukoglu, Gokcan Aman, Afsin İpekci

**Affiliations:** ^1^ Istanbul University-Cerrahpasa, Cerrahpasa Faculty of Medicine, Department of Emergency Medicine, Kocamustafapasa st. No: 53 Fatih, İstanbul, 34098, Turkey, istanbul.edu.tr; ^2^ Istanbul University-Cerrahpasa, Cerrahpasa Faculty of Medicine, Department of Cardiology, Kocamustafapasa st. No: 53 Fatih, İstanbul, 34098, Turkey, istanbul.edu.tr; ^3^ Istanbul University-Cerrahpasa, Cerrahpasa Faculty of Medicine, Department of Biochemistry, Kocamustafapasa st. No: 53 Fatih, İstanbul, 34098, Turkey, istanbul.edu.tr; ^4^ Northampton General Hospital, Department of Accidents and Emergency, Cliftonville, Northampton, NN1 5BD, UK, nhs.uk

**Keywords:** copeptin, echocardiography, emergency medicine, H-FABP, pulmonary embolism

## Abstract

**Background:**

Pulmonary embolism (PE) remains a major diagnostic and prognostic challenge in emergency departments due to its heterogeneous clinical presentation and potentially high mortality. Although echocardiography and conventional cardiac biomarkers are commonly used in clinical practice, the additional prognostic value of novel biomarkers such as copeptin and heart‐type fatty acid‐binding protein (H‐FABP) requires further investigation.

**Methods:**

This exploratory prospective study (March 2021–September 2022) included adult patients (≥ 18 years) diagnosed with acute PE confirmed by radiological imaging in the emergency department. Patients with chronic/unconfirmed PE, incomplete data, or those referred from external centers were excluded. The primary analysis compared survivors and nonsurvivors at 30 days to evaluate the prognostic value of copeptin, H‐FABP, and echocardiographic parameters. Transthoracic echocardiography (TTE) was performed after diagnosis to assess right heart function and pulmonary artery pressure (PAP), right ventricular/left ventricular ratio (RV/LV), right and left ventricular end‐diastolic diameters (RV‐EDD and LV‐EDD), and tricuspid annular plane systolic excursion (TAPSE). Biomarkers (troponin, pro‐BNP, D‐dimer, copeptin, and H‐FABP) were analyzed using standard laboratory assays and enzyme‐linked immunosorbent assay (ELISA). The primary outcome was 30‐day mortality.

**Results:**

A total of 88 patients were analyzed. Although H‐FABP and copeptin levels were higher in nonsurvivors, these differences were not statistically significant for predicting 30‐day mortality. Echocardiographic parameters—particularly PAP, RV‐EDD, and RV/LV—were significantly associated with the severity of right ventricular dysfunction and clinical outcomes (*p* < 0.05). In multivariable logistic regression analysis copeptin, H‐FABP, PESI score, ICU admission, RV‐EDD, and LV‐EDD remained independently associated with 30‐day mortality. Cut‐off values were determined by ROC analysis using the Youden index.

**Conclusions:**

Although biomarkers were not significant as continuous variables, exploratory categorized analysis based on cut‐off values suggested a potential association with mortality. Rapid bedside echocardiography combined with appropriate biomarker testing may improve risk stratification in patients with PE in emergency settings, while larger studies are needed to clarify the role of these biomarkers.

## 1. Introduction

Pulmonary embolism (PE) is a serious cardiovascular condition and remains one of the most critical emergencies encountered in the emergency department (ED). Both the diagnosis and prognostic evaluation of PE can be challenging because of its highly variable clinical presentation and the substantial risk of mortality. Reported mortality rates vary widely, ranging from less than 1% in patients with preserved right ventricular function and normal troponin levels to more than 60% in cases presenting with cardiac arrest [1].

A variety of biomarkers have been investigated to assist with risk stratification and prognostic assessment in PE. Among these, troponin, pro‐brain natriuretic peptide (pro‐BNP), copeptin, heart‐type fatty acid‐binding protein (H‐FABP), and D‐dimer are commonly used in clinical practice [2, 3]. In addition to laboratory biomarkers, transthoracic echocardiography (TTE) plays an important role as a bedside imaging modality, allowing rapid assessment of right ventricular function, estimation of pulmonary artery pressure (PAP), and identification of hemodynamic compromise, particularly in unstable or intermediate‐risk patients [3, 4].

While the prognostic significance of copeptin and H‐FABP has been examined in several cardiovascular disorders, limited data exist regarding how these biomarkers perform when evaluated alongside echocardiographic indicators of right ventricular dysfunction.

Therefore, the present study aimed to compare survivors and nonsurvivors among patients diagnosed with acute PE in the ED by simultaneously evaluating novel biomarkers (copeptin and H‐FABP) and TTE parameters. Through this combined approach, we sought to clarify their relative prognostic contribution in predicting 30‐day mortality and right ventricular dysfunction in patients presenting with acute PE.

## 2. Materials and Methods

This study was conducted after obtaining ethical approval from the Istanbul University‐Cerrahpaşa Rectorate Clinical Research Ethics Committee (Approval Date: March 10, 2021; Approval Number: 49434). Written informed consent was obtained from all participants, and the study was performed in accordance with the principles of the Declaration of Helsinki.

Data were collected between March 11, 2021, and September 30, 2022, from patients admitted to the Emergency Department of Istanbul University‐Cerrahpaşa, Cerrahpaşa Faculty of Medicine, who were diagnosed with PE.

This study was supported by the Istanbul University‐Cerrahpaşa Scientific Research Projects Coordination Office (BAP; Project No: TTU‐2021‐36152), which provided funding for laboratory assay kits used in the biomarker analyses.

### 2.1. Data Collection

Adult patients aged ≥ 18 years with radiologically confirmed PE diagnosed by either computed tomography pulmonary angiography (CTPA) or ventilation–perfusion (V/Q) scintigraphy were included in the study. Patients younger than 18 years of age, those who already had chronic PE, individuals with incomplete data, patients referred from external centers, and those who declined participation were excluded.

Collected data included patient demographics, comorbidities, presenting complaints, vital signs, clinical symptoms, physical examination findings, and electrocardiographic (ECG) results. The anatomical location of PE (main, lobar, segmental, or subsegmental) was determined based on radiological imaging findings.

Following confirmation of PE diagnosis by CTPA or V/Q scintigraphy, all patients underwent TTE performed by an experienced cardiologist within the first 24 h of admission to the ED. Standard parasternal long‐axis, parasternal short‐axis, apical four‐chamber, and subcostal views were obtained.

Echocardiographic parameters included pulmonary artery pressure (PAP), estimated from tricuspid regurgitation velocity using the modified Bernoulli equation with the addition of right atrial pressure; tricuspid annular plane systolic excursion (TAPSE), measured using M‐mode in the apical four‐chamber view, with values < 16 mm indicating right ventricular systolic dysfunction; the right ventricular to left ventricular (RV/LV) ratio measured at end‐diastole in the apical four‐chamber view, with values > 1.0 considered abnormal; right and left ventricular end‐diastolic diameters (RV‐EDD and LV‐EDD) measured in the parasternal long‐axis view; and left ventricular ejection fraction (EF), calculated using the modified Simpson’s biplane method. Pulmonary hypertension was defined as PAP ≥ 30 mmHg [3, 5].

Right ventricular dysfunction was further evaluated using additional echocardiographic indicators, including right ventricular outflow tract (RVOT) enlargement, reduced contractility of the right ventricular free wall relative to the apex (McConnell’s sign), and reduced TAPSE values. Patients were categorized according to the severity of right heart failure based on these echocardiographic findings. The involvement of pathological findings in at least two parameters was classified as advanced right heart failure, the presence of a single parameter as moderate right heart failure, and the absence of pathological findings as mild/none right heart failure [3, 5].

Admission to either the general ward or the intensive care unit (ICU) was recorded for all patients. Thirty‐day mortality was assessed using hospital records and the national Death Notification System (https://obs.saglik.gov.tr). The primary outcome was 30‐day mortality. Secondary outcomes included echocardiographic markers of right ventricular dysfunction and clinical severity indicators such as ICU admission.

This study was designed as an exploratory prospective observational study.

### 2.2. Analysis of Serum/Blood Samples

Blood samples were obtained immediately after confirmation of the PE diagnosis and prior to the initiation of treatment. Levels of troponin, pro‐BNP, and D‐dimer were analyzed using standard assay kits available in the Emergency Biochemistry Unit of Cerrahpaşa Faculty of Medicine.

For further biomarker analysis, venous blood samples were collected into serum tubes and centrifuged at 3000 rpm for 20 min to obtain serum samples. The separated serum was subsequently stored at −80°C until analysis.

Serum levels of H‐FABP and copeptin were measured using commercially available enzyme‐linked immunosorbent assay (ELISA) kits (Lot Nos. E4045hu and E1129Hu; BT Lab, China). All reagents were allowed to equilibrate to room temperature prior to analysis in accordance with the manufacturer’s instructions.

### 2.3. Statistical Analysis

All statistical analyses were performed using IBM SPSS Statistics version 26 (IBM Corp., Armonk, NY, USA). Descriptive statistics were presented as frequencies and percentages for categorical variables, medians with interquartile ranges for discrete variables, and means with standard deviations for continuous variables. The normality of distribution for continuous variables was assessed using the Kolmogorov–Smirnov test.

Categorical variables were compared using Pearson’s chi‐square test or Fisher’s exact test, as appropriate. For continuous variables with normal distribution, comparisons between two groups were performed using the Student’s *t*‐test, while comparisons among three or more groups were conducted using one‐way analysis of variance (ANOVA) followed by Tukey’s HSD post hoc test. For non‐normally distributed variables, the Mann–Whitney *U* test (two groups) and the Kruskal–Wallis test (three or more groups) were used.

Risk estimates and odds ratios (OR) were calculated using chi‐square analysis. A two‐sided *p* value < 0.05 was considered statistically significant. Cut‐off values for copeptin and H‐FABP were determined using the Youden index. The optimal thresholds were identified as 3.57 ng/mL for copeptin and 2.60 ng/mL for H‐FABP. Based on these values, copeptin and H‐FABP were categorized as high or low for subsequent regression analyses. Multivariable logistic regression analyses were performed to identify factors associated with 30‐day mortality. Receiver operating characteristic (ROC) curve analysis was performed to determine the diagnostic performance of biomarkers, and optimal cut‐off values were identified using the Youden index.

## 3. Results

Between March 2021 and September 2022, a total of 108 patients presented to the ED and were diagnosed with PE based on imaging studies. Of these, 88 patients met the inclusion criteria and were included in the final analysis. Twenty patients were excluded due to incomplete echocardiographic or laboratory data or because they declined participation.

Among the included patients, 39 were male (44.32%) and 49 were female (55.68%). The most frequently represented age group was 60–70 years (*n* = 28) (Table 1).

**TABLE 1:** Distribution of patients according to age at referral time.

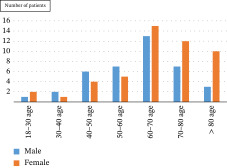

The demographic characteristics, clinical findings, imaging results, and echocardiographic parameters of the study population are summarized in Table 2. Information regarding mortality rates and patient follow‐up settings is also presented in the same table.

**TABLE 2:** Demographic, clinical, imaging and echocardiographic findings of patients with pulmonary embolism.

**Gender distribution**	***n***	**%**

Male	39	(44.32%)
Female	49	(55.68%)

**Comorbidities**	***n***	**%**

Cardiac etiologies	51	(48.86%)
Malignancy	31	(35.23%)

**Presenting symptoms**	***n***	**%**

Shortness of breath	43	(48.86%)
Chest pain	16	(18.18%)

**Electrocardiographic findings**	***n***	**%**

Normal sinus rhythm	47	(53.41%)
Atrial fibrillation	13	(14.77%)
Sinus tachycardia	20	(22.73%)

**Embolism localization**	***n***	**%**

Main branches	19	(21.59%)
Lobar branches	26	(29.55%)
Segmental branches	31	(35.23%)
Subsegmental branches	12	(13.64%)

**Echocardiographic findings**	**(Mean ± SD)**	

EF (%)	51.57 ± 8.04	
TAPSE (mm)	22.03 ± 3.54	
PAP (mmHg)	36.20 ± 13.17	
RV‐EDD (mm)	37.38 ± 5.04	
LV‐EDD (mm)	43.39 ± 6.08	
RV/LV ratio	0.87 ± 0.13	

**Pulmonary hypertension**	***n***	**%**

Absent	75	(85.23%)
Present	13	(14.77%)

**Systolic heart failure**	***n***	**%**

Absent	69	(78.41%)
Present	19	(21.59%)

**Type of systolic heart failure**	***n***	**%**

Right	10	(11.36%)
Left	7	(7.95%)
Global	2	(2.27%)

**TAPSE values**	***n***	**%**

< 16 mm	6	(6.82%)
≥ 16 mm	82	(93.18%)

**PAP values**	***n***	**%**

20–25 mmHg	13	(14.77%)
25–30 mmHg	22	(25.00%)
≥ 30 mmHg	53	(60.23%)

**EF values**	***n***	**%**

< 40%	7	(7.95%)
40%–50%	23	(26.14%)
≥ 50%	58	(65.91%)

**RV/LV ratio**	***n***	**%**

≤ 1	75	(85.23%)
> 1	13	(14.77%)

**Follow-up location**	***n***	**%**

Intensive care unit	18	(20.45%)
Service wards	69	(78.41%)
Emergency department	1	(1.14%)

**30-day mortality**	***n***	**%**

Mortality observed	20	(22.73%)
Mortality not observed	68	(77.27%)

Abbreviations: EDD, end diastolic diameter; EF, ejection fraction; PAP, pulmonary arterial pressure; RV/LV, right ventricle/left ventricle; TAPSE, tricuspid annular plane systolic excursion.

When 30‐day mortality was analyzed in relation to age and vital signs, both the mean age and respiratory rate were significantly higher in patients who died compared with survivors (Table 3).

**TABLE 3:** Relation of age and vital signs with mortality.

30‐day mortality	None (*n* = 68) mean ± SD	Observed (*n* = 20) mean ± SD	*p*
Age	62.65 ± 17.12	69.00 ± 7.99	**0.023**
SBP (mmHg)	135 ± 28	123 ± 26	0.093
DBP (mmHg)	82 ± 18	75 ± 20	0.145
Pulse (bpm)	97 ± 23	100 ± 27	0.648
sPO2 (%)	92 ± 6	86 ± 13	0.067
Temperature (°C)	36.52 ± 0.46	36.43 ± 0.28	0.397
Respiratory Rate	21.49 ± 5.96	24.80 ± 7.44	**0.042**

*Note:* sPO2: oxygen saturation. The statistically significant values have been highlighted in bold to improve readability and facilitate identification of significant findings.

Abbreviations: DBP, diastolic blood pressure; SBP, systolic blood pressure.

PESI scores were significantly higher among nonsurvivors compared with survivors (*p* < 0.001). In contrast, echocardiographic analysis revealed a significantly lower LV‐EDD in patients who died compared with survivors (*p* = 0.013) (Table 4).

**TABLE 4:** Relation of biomarkers and echocardiographic findings with mortality.

30‐day mortality	None (*n* = 68) mean ± SD	Observed (*n* = 20) mean ± SD	*p*
Troponin (ng/mL)	0.058 ± 0.151	0.064 ± 0.091	0.852
D‐dimer (mg/L)	7.40 ± 10.42	8.94 ± 11.80	0.575
Pro‐BNP (pg/mL)	2916 ± 6776	6892 ± 12577	0.188
H‐FABP (ng/mL)	4.14 ± 4.57	4.87 ± 3.68	0.515
Copeptin (ng/mL)	4.26 ± 3.91	4.07 ± 2.43	0.799
EF (%)	52.10 ± 7.65	49.75 ± 9.24	0.252
TAPSE (mm)	21.88 ± 3.59	22.55 ± 3.41	0.462
PAP (mmHg)	36.00 ± 13.46	36.90 ± 12.45	0.782
RV‐EDD (mm)	37.43 ± 5.35	37.20 ± 3.96	0.861
LV‐EDD (mm)	44.01 ± 6.57	41.25 ± 3.29	**0.013**
RV/LV	0.86 ± 0.14	0.90 ± 0.08	0.102
PESI	99.52 ± 35.84	149.50 ± 44.34	**< 0.001**

*Note:* Pro‐BNP: pro‐brain natriuretic peptide. The statistically significant values have been highlighted in bold to improve readability and facilitate identification of significant findings.

Abbreviations: EDD, end diastolic diameter; EF, ejection fraction; H‐FABP, heart‐fatty acid‐binding protein; PAP, pulmonary arterial pressure; PESI, pulmonary embolism severity index; RV/LV, right ventricle/left ventricle; TAPSE, tricuspid annular plane systolic excursion.

Mortality was significantly higher among patients admitted to the ICU than among those managed in the general ward (Table 5).

**TABLE 5:** Mortality difference between service and ICU follow‐up.

		Mortality *n* (%)		
		None	Observed	*p*
Follow‐up	Service	62 (70.5%)	7 (8.0%)	**< 0.001**
	ICU	6 (6.8%)	13 (14.8%)	

*Note:* The statistically significant values have been highlighted in bold to improve readability and facilitate identification of significant findings.

Abbreviation: ICU, intensive care unit.

PAP, the RV/LV ratio, and RV‐EDD differed significantly between patients with mild or no right heart failure and those with advanced right heart failure. In addition, RV‐EDD values were significantly different when patients with mild or no right heart failure were compared with those with moderate right heart failure (Table 6).

**TABLE 6:** Analysis of right heart failure severity.

TTE Findings	Mild/none	Moderate	Advanced	*p*
PAP (mmHg)	33.16 ± 9.78	42.25 ± 14.51	59.67 ± 18.93	0.012**^a^**
TAPSE (mm)	22.14 ± 3.44	21.08 ± 3.85	22.67 ± 4.46	0.576
EF (%)	52.96 ± 6.22	45.50 ± 11.65	47.50 ± 12.55	0.121
RV/LV	0.85 ± 0.11	0.88 ± 0.15	1.09 ± 0.16	< 0.001**^a^**
RV‐EDD (mm)	36.41 ± 4.49	39.17 ± 2.48	45.00 ± 7.80	0.010**^a^** ^**,**^ **^b^**
LV‐EDD (mm)	43.19 ± 5.71	45.67 ± 8.73	41.17 ± 2.56	0.280

*Note:* The statistically significant values have been highlighted in bold to improve readability and facilitate identification of significant findings.

Abbreviations: EDD, end diastolic diameter; EF, ejection fraction; PAP, pulmonary arterial pressure; RV/LV, right ventricle/left ventricle; TAPSE, tricuspid annular plane systolic excursion.

^a^Significance of advanced heart failure compared to mild/none heart failure.

^b^Significance of moderate heart failure compared to mild/none heart failure.

**FIGURE 1:**
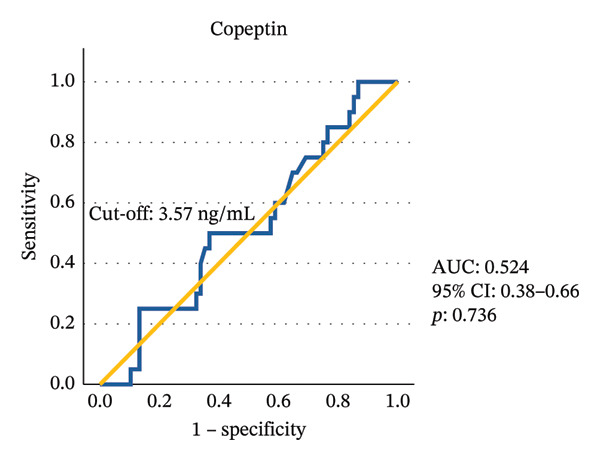
ROC curve analysis of copeptin for 30‐day mortality.

**FIGURE 2:**
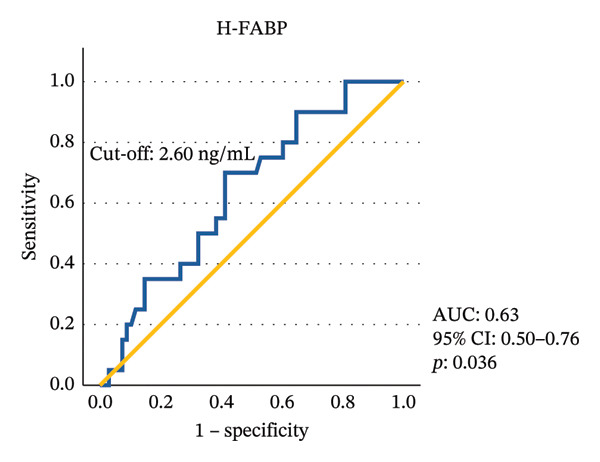
ROC curve analysis of H‐FABP for 30‐day mortality.

Using the Youden index, the optimal cut‐off values were identified as 3.57 ng/mL for copeptin and 2.60 ng/mL for H‐FABP, particularly area under curve (AUC): 0.524 (95% CI: 0.38–0.66, *p* = 0.736); AUC: 0.63 (95% CI: 0.50–0.76, *p* = 0.036). Based on these thresholds, copeptin and H‐FABP were categorized as high or low values. ROC analysis demonstrated an AUC of 0.524 for copeptin and 0.630 for H‐FABP in predicting 30‐day mortality (Figures 1 and 2).

In multivariable logistic regression analysis, copeptin and H‐FABP remained independently associated with mortality (*p* = 0.028 and *p* = 0.007, respectively). Other significant predictors included PESI score, ICU admission, RV‐EDD, and LV‐EDD (Table 7). TAPSE (< 16 mm), pulmonary hypertension (PAP ≥ 30 mmHg), and RV/LV ratio > 1 were used to define right ventricular dysfunction and were included in subsequent logistic regression analyses for 30‐day mortality.

**TABLE 7:** Multivariate logistic regression analysis of laboratory, echocardiographic variables, and severity parameters for 30‐day mortality.

Variable	*B*	OR (95% CI)	*p*
Copeptin	3.14	23.22 (1.41–381.51)	**0.028**
H‐FABP	2.52	12.51 (1.97–79.26)	**0.007**
Troponin	2.77	4.58 (1.65–28.63)	0.088
D‐dimer	0.01	1.01 (0.97–1.05)	0.574
Pro‐BNP	0.00	1.00 (1.00–1.00)	0.138
EF	−0.01	0.99 (0.90–1.08)	0.831
PHT	0.585	1.79 (0.45–4.84)	0.639
RV/LV	2.13	3.46 (0.93–17.01)	0.091
TAPSE	−19.02	0.00	0.999
RV‐EDD	0.18	1.19 (1.00–1.42)	**0.043**
LV‐EDD	−0.26	0.76 (0.62–0.94)	**0.012**
PESI score	0.02	1.02 (1.00–1.04)	**0.037**
ICU admission	4.05	57.52 (2.71–1217.62)	**0.009**

*Note:* Pro‐BNP: pro‐brain natriuretic peptide, PHT: pulmonary hypertension. Categorical values (copeptin: ≥ 3.57 ng/mL, H‐FABP: ≥ 2.60 ng/mL, TAPSE: < 16 mm, RV/LV > 1.0, PHT = PAP ≥ 30 mmHg). The statistically significant values have been highlighted in bold to improve readability and facilitate identification of significant findings.

Abbreviations: EDD, end diastolic diameter; EF, ejection fraction; H‐FABP, heart‐fatty acid‐binding protein; ICU, intensive care unit; PAP, pulmonary arterial pressure; PESI, pulmonary embolism severity index; RV/LV, right ventricle/left ventricle; TAPSE, tricuspid annular plane systolic excursion.

## 4. Discussion

Our demographic and clinical findings also provide additional context for interpreting the biomarker results. Female patients constituted a slightly larger proportion of the study population than males, and mortality rates were also higher among women. In addition, hypertension was the most common comorbidities, followed by malignancy.

In the present study, PAP, RV/LV ratio, and RV‐EDD were significantly associated with right ventricular dysfunction severity in acute PE, supporting the established prognostic role of echocardiographic assessment. Cho et al. showed that patients with a RV/LV > 1 had higher rates of ICU admission [6], while Hsiao et al. demonstrated significantly elevated PAP and impaired right ventricular function in patients with PE compared with healthy controls [7]. Overall, both our findings and prior evidence support the central role of echocardiography in early PE severity assessment.

In our study, copeptin and H‐FABP were not associated with mortality as continuous variables; however, categorization using predefined cut‐off values revealed significant associations.

Copeptin levels showed no significant differences across heart failure severity groups or between survivors and nonsurvivors. Usul et al. found higher copeptin levels in patients with right ventricular dilation [8], whereas Ozmen et al. reported associations with mortality and right ventricular failure [9]. The discrepancy may be related to differences in patient selection, comorbidity burden, study population heterogeneity, or relatively limited sample size of our cohort.

Although patients with advanced right heart failure had higher mean H‐FABP levels, this difference was not statistically significant. Lauque et al. reported increased risk of adverse outcomes with H‐FABP > 6 ng/mL [10], and Dellas et al. showed associations with short‐term complications [11]. Boscheri et al. similarly reported worse outcomes in patients with elevated H‐FABP levels [12]. The trend observed in our cohort may suggest a potential association that requires confirmation in larger studies.

Kalkan et al. reported that copeptin levels > 4.84 ng/mL predicted PE with 68.1% sensitivity and 83.7% specificity, and copeptin has also been identified as an independent predictor of confirmed PE in multivariable analysis (OR 1.836, 95% CI 1.17–2.88) [13]. In contrast, Parlak et al. found no significant difference in copeptin levels between patients with PE and healthy controls [14]. For H‐FABP, Dellas et al. and Puls et al. identified a cut‐off value of 6 ng/mL using ROC analysis in normotensive PE cohorts [11, 15], and elevated H‐FABP levels (≥ 6 ng/mL) were also reported by Dellas as an independent predictor of complicated 30‐day outcomes in PE [11]. In the exploratory logistic regression analysis based on cut‐off values (Table 7), both copeptin and H‐FABP were significantly associated with increased 30‐day mortality. Elevated copeptin levels (≥ 3.57 ng/mL) were associated with higher mortality risk (*p* = 0.028), and elevated H‐FABP levels (≥ 2.60 ng/mL) showed a similar association (*p* = 0.007). However, the wide confidence intervals suggest that these findings should be interpreted cautiously.

Beyond copeptin and H‐FABP, other circulating markers have also been investigated in PE. Recent data suggest that endothelial PAS domain protein‐1 (EPAS‐1) may have potential clinical relevance. Icli et al. reported significantly higher EPAS‐1 levels in PE patients than in controls (3.6 ± 1.42; 1.57 ± 0.45, *p* < 0.001) and found a positive correlation with PESI scores, with persistently elevated levels in patients who died [16]. Alsancak et al. reported that EPAS‐1 levels were significantly higher in patients with systemic lupus erythematosus compared with controls and showed a moderate positive correlation with right ventricular systolic motion (*p* = 0.007) [17]. These observations support the broader concept that emerging biomarkers may have potential prognostic and diagnostic value in PE

Another important aspect of our study is the timing of echocardiographic evaluation. Because echocardiography was performed within the first 24 h after admission, early initiation of anticoagulation or supportive treatment may have influenced right ventricular measurements. Future investigations should further examine how the timing of imaging and treatment initiation affects both echocardiographic and biomarker‐based risk assessment.

Several additional factors may have influenced the observed associations and may confuse treatment‐related results, including baseline disease severity, variability in treatment strategies, initiation of anticoagulation therapy or antithrombolytic agents, and the timing of biomarker sampling and echocardiographic assessment. In addition, underlying comorbid conditions—particularly cardiovascular disease, malignancy, and renal dysfunction—may have affected both biomarker levels and clinical outcomes, thereby acting as potential confounders.

The additional prognostic contribution of copeptin and H‐FABP appears limited in a heterogeneous ED population. Therefore, the lack of statistical significance in our study should not be interpreted as the absence of prognostic value, but rather as a reflection of the limited sample size and event number. Larger multicenter studies involving more homogeneous patient groups are needed to better define their clinical utility.

### 4.1. Limitations

Although no statistically significant association was observed between copeptin or H‐FABP levels and 30‐day mortality, the possibility of a Type II error cannot be excluded. The relatively small sample size and the limited number of mortality events may have reduced the statistical power to detect smaller but clinically relevant differences, particularly in analyses evaluating biomarker performance. Therefore, the absence of statistical significance should be interpreted with caution, and larger multicenter studies are needed to further clarify the prognostic value of these biomarkers.

In addition, certain practical limitations may affect the use of cardiac biomarkers in emergency settings. Compared with echocardiography, which provides immediate bedside information, laboratory‐based biomarker analyses may require longer processing times.

Finally, our ED serves as a tertiary referral center, and many critically ill patients are transferred from other hospitals before a definitive diagnosis is established. The high physiological stress and potential pre‐existing cardiac injury observed in these patients may also influence biomarker levels, potentially limiting the clinical applicability of biomarker‐based risk assessment in this setting. Given the relatively small sample size and limited number of events, the present study should be considered exploratory in nature. Furthermore, this was a single‐center study, which may limit the generalizability of the findings to other populations and healthcare settings.

## 5. Conclusion

Although copeptin and H‐FABP were not significantly associated with 30‐day mortality when analyzed as continuous variables, exploratory analyses using categorized values suggested a potential association. By prospectively evaluating novel biomarkers together with echocardiographic parameters, our study offers a clinically relevant perspective on early risk stratification in patients presenting with acute PE. These findings should be interpreted with caution given the limited sample size and number of events. Larger multicenter studies are required to further clarify the prognostic value of these biomarkers. Rapid bedside echocardiography remains a valuable tool for risk stratification in patients with acute PE in the emergency setting.

**Nomenclature**
PE: Pulmonary embolism
pro‐BNP: Pro‐brain natriuretic peptide
H‐FABP: Heart‐type fatty acid‐binding protein
CT: Computerized tomography
ED: Emergency department
EPAS‐1: Endothelial PAS domain protein‐1
ELISA: Enzyme‐linked immunosorbent assay
PAP: Pulmonary artery pressure
RV/LV: Right ventricle/left ventricle
CTPA: Computed tomography pulmonary angiography
V/Q: Ventilation–perfusion
ECG: Electrocardiography
TAPSE: Tricuspid annular plane systolic excursion
TTE: Transthoracic echocardiography
AF: Atrial fibrillation
EF: Ejection fraction
PHT: Pulmonary hypertension
ICU: Intensive care unit
EDD: End diastolic diameter
SBP: Systolic blood pressure
DBP: Diastolic blood pressure
sPO2: Oxygen saturation
LV‐EDD: Left ventricular end‐diastolic diameter
RV‐EDD: Right ventricular end‐diastolic diameter
DVT: Deep vein thrombosis
VTE: Venous Thromboembolism
PESI: Pulmonary embolism severity index
RVEF: Right ventricular ejection fraction
AUC: Area under curve
ROC: Receiver operating characteristic

## Funding

This research was funded by the Istanbul University‐Cerrahpaşa, Scientific Research Projects Coordination Office (Project No: TTU‐2021‐36152).

## Conflicts of Interest

The authors declare no conflicts of interest.

## Supporting Information

Additional supporting information can be found online in the Supporting Information section.

## Supporting information


**Supporting Information** STROBE‐checklist(2)‐v4‐Hüseyin Furkan Küçükbezirci

## Data Availability

The data that support the findings of this study are not publicly available due to patient privacy and ethical restrictions.
