# Supplementary material for: Prognostic Role of Copeptin, H‐FABP, and TTE in Pulmonary Embolism in the Emergency Department
Source: Emerg Med Int. 2026 Jun 29;2026:9544238. doi: 10.1155/emmi/9544238 (PMC13312803; doi:10.1155/emmi/9544238)
Supplement: Supplementary file 1 — Supporting Information STROBE‐checklist(2)‐v4‐Hüseyin Furkan Küçükbezirci [file EMMI-2026-9544238-s001.docx]

STROBE Statement—checklist of items that should be included in reports of observational studies

|  | Item No. | Recommendation | Page  No. | Relevant text from manuscript |
| --- | --- | --- | --- | --- |
| **Title and abstract** | 1 | (*a*) Indicate the study’s design with a commonly used term in the title or the abstract | 3, 7 | Study design is stated in the Abstract as a “prospective single-center study.” And “This study was designed as an exploratory prospective observational study.” was stated in Materials and Methods |
|  |  | (*b*) Provide in the abstract an informative and balanced summary of what was done and what was found | 3 | The Abstract provides a structured and balanced summary of the study, including methods, results, and conclusions, with added information on logistic regression analyses. |
| Introduction | | | |  |
| Background/rationale | 2 | Explain the scientific background and rationale for the investigation being reported | 4 | The Introduction explains the clinical importance of pulmonary embolism and the rationale for evaluating biomarkers and echocardiographic parameters. |
| Objectives | 3 | State specific objectives, including any prespecified hypotheses | 4-5 | The study objectives are clearly stated in the Introduction, focusing on the prognostic value of copeptin, H-FABP, and echocardiographic parameters in predicting 30-day mortality. |
| Methods | | | |  |
| Study design | 4 | Present key elements of study design early in the paper | 5 | Key elements of the study design are presented at the Methods section as a prospective single-center study and exploratory prospective observational study. |
| Setting | 5 | Describe the setting, locations, and relevant dates, including periods of recruitment, exposure, follow-up, and data collection | 5 | The setting, location, and study period (March 2021–September 2022) are clearly described in the Methods section, including patient recruitment and follow-up. |
| Participants | 6 | (*a*) *Cohort study*—Give the eligibility criteria, and the sources and methods of selection of participants. Describe methods of follow-up  *Case-control study*—Give the eligibility criteria, and the sources and methods of case ascertainment and control selection. Give the rationale for the choice of cases and controls  *Cross-sectional study*—Give the eligibility criteria, and the sources and methods of selection of participants | 5 | Eligibility criteria, patient selection, and follow-up methods are clearly described in the Data Collection section. |
|  |  | (*b*) *Cohort study*—For matched studies, give matching criteria and number of exposed and unexposed  *Case-control study*—For matched studies, give matching criteria and the number of controls per case | 5 | Not applicable (no matching design was used). |
| Variables | 7 | Clearly define all outcomes, exposures, predictors, potential confounders, and effect modifiers. Give diagnostic criteria, if applicable | 3, 6, 7, 8 | The primary outcome was 30-day mortality. Secondary outcomes included echocardiographic markers of right ventricular dysfunction and clinical severity indicators such as ICU admission. Primary outcome was all-cause mortality, assessed via the hospital follow-up system. Clinical follow-up included whether patients were monitored in the ward or ICU. Echocardiographic parameters, particularly findings of right heart failure (e.g., right ventricular dysfunction, dilation, and pressure overload), were evaluated as predictors. Cut-off values were determined using the Youden Index from ROC analysis. Potential confounders included age, comorbidities, and baseline clinical status, while ICU admission was considered a potential effect modifier. Right heart failure was defined in Materials and Methods. |
| Data sources/ measurement | 8* | For each variable of interest, give sources of data and details of methods of assessment (measurement). Describe comparability of assessment methods if there is more than one group | 6-7 | Data sources and measurement methods, including TTE parameters and ELISA-based biomarker analysis, are described in the Methods section. |
| Bias | 9 | Describe any efforts to address potential sources of bias | 19-20 | Potential sources of bias, including sample size and referral bias, are addressed in the Limitations section. Additionally, treatment-related results were defined in the Discussion section. |
| Study size | 10 | Explain how the study size was arrived at | 5-6, 19 | The study size was determined based on the number of eligible patients meeting the predefined inclusion criteria during the study period, as described in the Methods section, and was also influenced by resource-based constraints related to biomarker assays that explained in the Limitations section |

Continued on next page

| Quantitative variables | 11 | Explain how quantitative variables were handled in the analyses. If applicable, describe which groupings were chosen and why | 7-8 | Quantitative variables were analysed using appropriate statistical tests and categorized using cut-off values determined by the Youden index, specially copeptin and H-FABP. |
| --- | --- | --- | --- | --- |
| Statistical methods | 12 | (*a*) Describe all statistical methods, including those used to control for confounding | 7-8 | Statistical methods, including multivariable logistic regression analyses to control for confounding, are described in the Statistical Analysis section. |
|  |  | (*b*) Describe any methods used to examine subgroups and interactions | 6-7 | Subgroup analyses were performed based on right heart failure severity, as described in the Materials and Methods section. |
|  |  | (*c*) Explain how missing data were addressed | 5-6 | Patients with missing data were excluded from the analysis, as stated in the Methods section. |
|  |  | (*d*) *Cohort study*—If applicable, explain how loss to follow-up was addressed  *Case-control study*—If applicable, explain how matching of cases and controls was addressed  *Cross-sectional study*—If applicable, describe analytical methods taking account of sampling strategy | 5-7 | Follow-up was completed for all included patients using hospital records and the national death registry; loss to follow-up was not observed. |
|  |  | (*e*) Describe any sensitivity analyses | (-) | Sensitivity analyses were not performed. |
| Results | | | | |
| Participants | 13* | (a) Report numbers of individuals at each stage of study—eg numbers potentially eligible, examined for eligibility, confirmed eligible, included in the study, completing follow-up, and analysed | 8 | The number of patients at each stage (108 assessed, 88 included) is clearly reported in the Results section. |
|  |  | (b) Give reasons for non-participation at each stage | 8 | Reasons for exclusion, incomplete echocardiographic or laboratory data and refusal to participate, are described in the Results section. |
|  |  | (c) Consider use of a flow diagram | (-) | A flow diagram was not included. |
| Descriptive data | 14* | (a) Give characteristics of study participants (eg demographic, clinical, social) and information on exposures and potential confounders | 9-11 | Demographic and clinical characteristics of participants are presented in Table 2. |
|  |  | (b) Indicate number of participants with missing data for each variable of interest |  | Patients with missing data were excluded; detailed per-variable missing data were not reported. |
|  |  | (c) *Cohort study*—Summarise follow-up time (eg, average and total amount) | 7 | A 30-day follow-up period was applied for all patients. |
| Outcome data | 15* | *Cohort study*—Report numbers of outcome events or summary measures over time | 8-16 | The number of outcome events (30-day mortality and ICU admission and echocardiographic parameters) is clearly reported in the Results section. |
|  |  | *Case-control study—*Report numbers in each exposure category, or summary measures of exposure |  |  |
|  |  | *Cross-sectional study—*Report numbers of outcome events or summary measures |  |  |
| Main results | 16 | (*a*) Give unadjusted estimates and, if applicable, confounder-adjusted estimates and their precision (eg, 95% confidence interval). Make clear which confounders were adjusted for and why they were included | 15-16 | Multivariabke logistic regression analysis estimates with odds ratios and 95% confidence intervals are reported in Tables 7 |
|  |  | (*b*) Report category boundaries when continuous variables were categorized | 6-7, 15 | Continuous variables were categorized using cut-off values determined by the Youden index (copeptin: 3.57, H-FABP: 2.60), and clinically established thresholds (e.g., TAPSE <16 mm, RV/LV>1.0, PHT = PAP ≥30 mmHg), as presented in Table 7. |
|  |  | (*c*) If relevant, consider translating estimates of relative risk into absolute risk for a meaningful time period |  | Not applicable. |

Continued on next page

| Other analyses | 17 | Report other analyses done—eg analyses of subgroups and interactions, and sensitivity analyses | 14 | Additional analyses include subgroup comparisons based on right heart failure severity. |
| --- | --- | --- | --- | --- |
| Discussion | | | | |
| Key results | 18 | Summarise key results with reference to study objectives | 19-20 | The Discussion and Conclusion summarise the main findings in relation to the study objectives, including biomarker and echocardiographic associations with 30-day mortality. |
| Limitations | 19 | Discuss limitations of the study, taking into account sources of potential bias or imprecision. Discuss both direction and magnitude of any potential bias | 19-20 | The Limitations section discusses sample size, limited mortality events, referral bias, and potential effects of comorbidities on biomarker interpretation. Additionally, treatment-related results were defined in the Discussion section. |
| Interpretation | 20 | Give a cautious overall interpretation of results considering objectives, limitations, multiplicity of analyses, results from similar studies, and other relevant evidence | 20 | The Discussion provides a cautious interpretation of the results in light of study objectives, limitations, multivariable analyses, and previous literature. |
| Generalisability | 21 | Discuss the generalisability (external validity) of the study results | 19-20 | Generalisability is addressed in the Limitations section by noting the single-center design and tertiary referral setting. |
| Other information | |  | | |
| Funding | 22 | Give the source of funding and the role of the funders for the present study and, if applicable, for the original study on which the present article is based | 5 | The source of funding is clearly stated in the Materials and Methods section, and the funders had no role in study design, data collection, analysis, or interpretation. |

*Give information separately for cases and controls in case-control studies and, if applicable, for exposed and unexposed groups in cohort and cross-sectional studies.

**Note:** An Explanation and Elaboration article discusses each checklist item and gives methodological background and published examples of transparent reporting. The STROBE checklist is best used in conjunction with this article (freely available on the Web sites of PLoS Medicine at http://www.plosmedicine.org/, Annals of Internal Medicine at http://www.annals.org/, and Epidemiology at http://www.epidem.com/). Information on the STROBE Initiative is available at www.strobe-statement.org.
